# Supplementary material for: Overexpression of MsRCI2D and MsRCI2E Enhances Salt Tolerance in Alfalfa (Medicago sativa L.) by Stabilizing Antioxidant Activity and Regulating Ion Homeostasis
Source: Int J Mol Sci. 2022 Aug 29;23(17):9810. doi: 10.3390/ijms23179810 (PMC9456006; doi:10.3390/ijms23179810)
Supplement: Supplementary file 1 [file ijms-23-09810-s001.zip › ijms-1874497-supplementary.pdf]

Supplementary Table S1

Gene Primer	Sequence of primer (5'-3')	Use for
MsRCI2D	S: ATGGGTTCTGAAACATTTGTGG<br>AS: GGTCATCCAACTAATACATAAATGGC	amplication and qPCR
MsRCI2E	S: ATGCCTTCAACTTGTGAAATTATCTGTGAG<br>AS: CTAGTATTTGTTTGCATACAAAGGACGCC	amplication and qPCR
MsRCI2A-qPCR	S: ATATTGAAAATGGGCACTGCTAC<br>AS: TGGAAGATGATGATATGGATCACT	qPCR
MsRCI2B-qPCR	S: ATGGGCACAGCTACATGCATC<br>AS: TCACTTGGTAATAGCATAGATAGCATAGA	qPCR
MsRCI2C-qPCR	S: ATGGGCACAGCTACCTTCG<br>AS: TCACTTAGTGATAATATAGATAGCATAGAT	qPCR
MsRCI2F-qPCR	S: ATGCCTTCACGTTGTGAAAT<br>AS: CTAGTATTGTGATTGTGCATACAAA	qPCR
Cu/Zn-SOD	S: TAATTGCTGATGCCAACG<br>AS: ACCACAGGCTAATCTTCCAC	qPCR
CAT	S: TTCTTCTTCTCCACCGTCCTCA<br>AS: TCCAAGAGAATTGGACCTCTGG	qPCR
GS	S: CTGTCAAATGCCCTTCAATA<br>AS: TGTTTCCTCCTCCTTCTCTC	qPCR
GR1	S: TGTGTCATTCGTGGTTGTG<br>AS: ACCCGCTATCTTTCCCTC	qPCR
ATPase	S:TTTGTTAATCAGGTAAAGCGATGTG<br>AS: TCCAAGTCAATGTCCGGTTG	qPCR
SOS1	S:TTGCTTACTACACTGCTCAAGA<br>AS: GCGATATAAGCAATCATTTCCCAA	qPCR
NHX1	S:CAATGCCGGGTTTCAAGTAAAG<br>AS:AGTAGCACCCGTGGTTATAATG	qPCR
HKT1	S:TTCATTGTCATGATGTATCTTCCAC<br>AS: CTGAGAGAATATAATACGATCCACTAGG	qPCR
GAPDH	S: GGCTGCATCAAGGAGGAAT<br>AS: TCCAAGCTCAGCCTCATCAAG	qPCR
Bar	S: ATGAGCCCAGAACGACGC<br>AS: TCAAATCTCGGTGACGGGC	PCR selection

Supplementary Figure S1

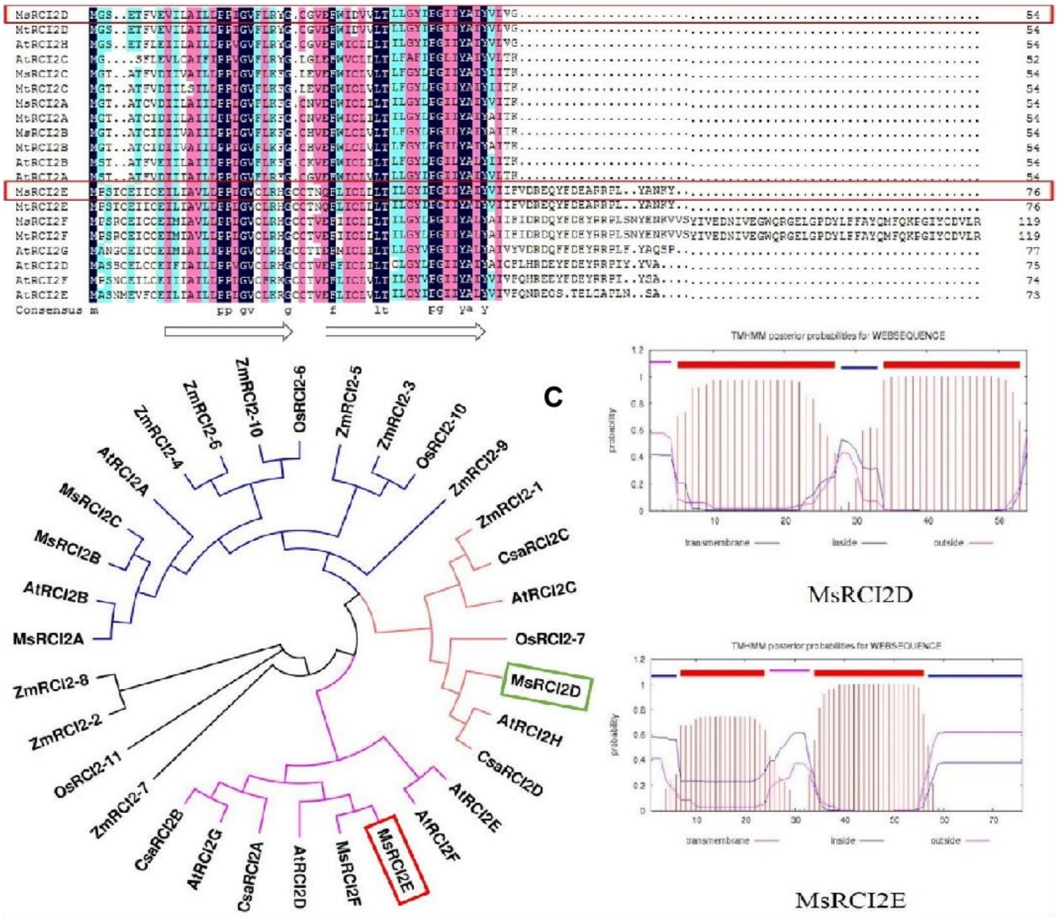

Sequence analysis of MsRCI2s proteins. (A) Amino acid sequence alignment of *RCI2s* genes in alfalfa and *Arabidopsis:*(B)Phylogenetic tree of RCI2 proteins in *Medicago sativa* L, *Arabidopsis thaliana*, *Zea mays*, rice and *Cucumis sativus* L.(C)Transmembrane domain analysis of MsRCI2D/E amimo acid sequence.

Supplementary Figure S2

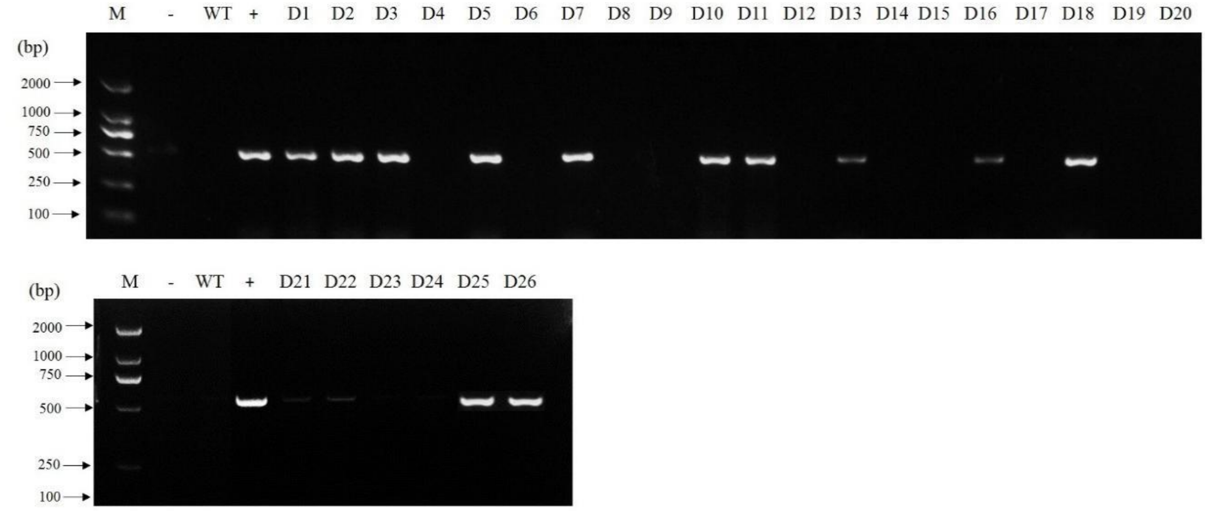

PCR detection of *MsRCI2D* transgenic hairy roots. M: Trans 2K DNA Marker; -: Negative control; +: Positive control; WT: Roots of non-transgenic lines; D1-D26: *MsRCI2D* transgenic hairy roots

Supplementary Figure S3

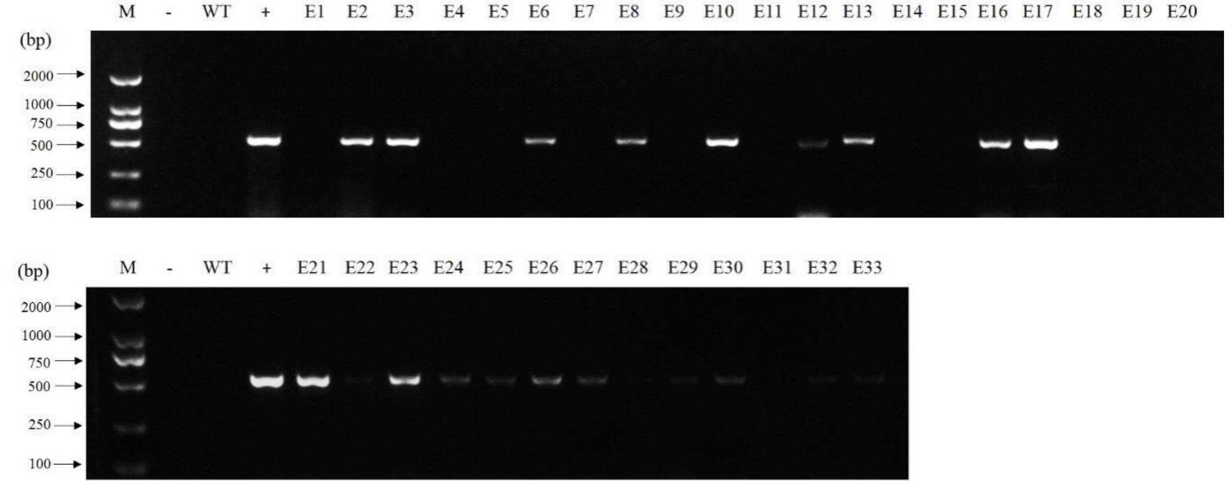

PCR detection of *MsRCI2E* transgenic hairy roots. M: Trans 2K DNA Marker; -: Negative control; +: Positive control; WT: Roots of non-transgenic lines; E1-E33: *MsRCI2E* transgenic hairy roots

Supplementary Figure S4

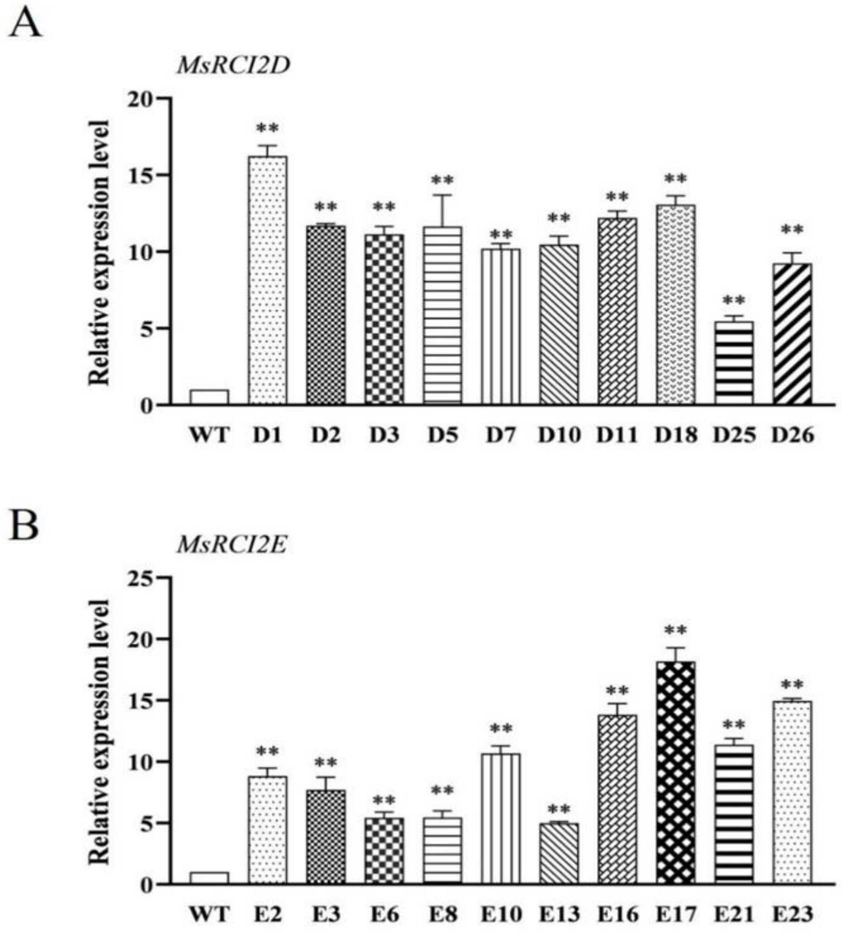

Relative expression levels of *MsRCI2D* (A) and *MsRCI2E* (B) in transgenic hairy roots. WT: Roots of non-transgenic lines; D1-D26: *MsRCI2D* transgenic hairy roots; E1-E35: *MsRCI2E* transgenic hairy roots. * indicates significant difference (p<0.05), ** indicates extremely significant difference (p<0.01)
